# *Clostridium difficile* colonization and antibiotics response in PolyFermS continuous model mimicking elderly intestinal fermentation

**DOI:** 10.1186/s13099-016-0144-y

**Published:** 2016-12-01

**Authors:** Sophie Fehlbaum, Christophe Chassard, Sophie Annick Poeker, Muriel Derrien, Candice Fourmestraux, Christophe Lacroix

**Affiliations:** 1 Laboratory of Food Biotechnology, Institute of Food, Nutrition and Health, ETH Zurich, Schmelzbergstrasse 7, 8092 Zurich, Switzerland; 2 Danone Nutricia Research, Palaiseau Cédex, France

**Keywords:** *Clostridium difficile*, Elderly gut microbiota, In vitro model, Intestinal fermentation, Antibiotics, Metronidazole, Ceftriaxone

## Abstract

**Background:**

*Clostridium difficile* (CD), a spore-forming and toxin-producing bacterium, is the main cause for antibiotic-associated diarrhea in the elderly. Here we investigated CD colonization in novel in vitro fermentation models inoculated with immobilized elderly fecal microbiota and the effects of antibiotic treatments.

**Methods:**

Two continuous intestinal PolyFermS models inoculated with different immobilized elder microbiota were used to investigate selected factors of colonization of CD in proximal (PC, model 1) and transverse-distal (TDC, model 1 and 2) colon conditions. Colonization of two CD strains of different PCR ribotypes, inoculated as vegetative cells (ribotype 001, model 1) or spores (ribotypes 001 and 012, model 2), was tested. Treatments with two antibiotics, ceftriaxone (daily 150 mg L^−1^) known to induce CD infection in vivo or metronidazole (twice daily 333 mg L^−1^) commonly used to treat CD, were investigated in TDC conditions (model 2) for their effects on gut microbiota composition (qPCR, 16S pyrosequencing) and activity (HPLC), CD spore germination and colonization, and cytotoxin titer (Vero cell assay).

**Results:**

CD remained undetected after inoculating vegetative cells in PC reactors of model 1, but was shown to colonize TDC reactors of both models, reaching copy numbers of up to log_10_ 8 mL^−1^ effluent with stable production of toxin correlating with CD cell numbers. Ceftriaxone treatment in TDC reactors showed only small effects on microbiota composition and activity and did not promote CD colonization compared to antibiotic-free control reactor. In contrast, treatment with metronidazole after colonization of CD induced large modifications in the microbiota and decreased CD numbers below the detection limit of the specific qPCR. However, a fast CD recurrence was measured only 2 days after cessation of metronidazole treatment.

**Conclusions:**

Using our in vitro fermentation models, we demonstrated that stable CD colonization in TDC reactors can be induced by inoculating CD vegetative cells or spores without the application of ceftriaxone. Treatment with metronidazole temporarily reduced the counts of CD, in agreement with CD infection recurrence in vivo. Our data demonstrate that CD colonized an undisturbed microbiota in vitro, in contrast to in vivo observations, thus suggesting an important contribution of host-related factors in the protection against CD infection.

**Electronic supplementary material:**

The online version of this article (doi:10.1186/s13099-016-0144-y) contains supplementary material, which is available to authorized users.

## Background

*Clostridium difficile* (CD) was first identified in the 1970s as the causative agent of antibiotic-associated pseudomembranous colitis and is now the leading cause of hospital-acquired diarrhea [1]. CD is a gram-positive anaerobic bacterium harboring several virulence factors such as the ability to form spores and produce toxins [2]. Interestingly, CD is part of the normal gut microbiota in 25–80% of infants but usually does not cause disease despite the finding that a significant fraction of the CD strains are toxin producers [3]. One of the main functions of an undisturbed gut microbiota is resistance against colonization of pathogens [4]. The disruption of the microbiota and thus the colonization resistance is usually the first step in the pathogenesis of CD infection (CDI). Following ingestion of CD spores, germination into the vegetative form is necessary for colonization in the gut, with subsequent toxin production leading to clinical manifestations [5].

Treatment with broad-spectrum antibiotics, such as ampicillin, clindamycin and third-generation cephalosporins (ceftriaxone, cefotaxime and ceftazidime) are considered to be main risk factors for CDI [6]. The elderly population is especially at risk of developing CDI, with CD colonization rates of up to 73% in inpatients above 65 years [7]. Standard therapy for CDI is antibiotic treatment with metronidazole or vancomycin. However, efficacy of these antibiotics is limited, with recurrence being observed in 20–40% of cases, mainly due to development of antibiotic resistances and a loss of gut barrier function that allows residual CD to re-colonize the colon after the antibiotic treatment is cancelled [1]. Indeed, the important role of an undisturbed microbiota that provides colonization resistance is reflected in the high success rate of fecal microbiota transplantation of up to circa 92% that is used in severe CDI cases [8].

Various animal models have been used to study the mechanisms underlying CDI and investigate antibiotic treatments. However, the use of animals is limited, primarily owing to ethical and practical reasons and notable inter-species differences in susceptibility to CDI. In vitro gut fermentation models represent an innovative technological platform consisting of multiple model designs that permit investigations, including survival or mechanistic studies on commensal–pathogen interactions and drug testing [9, 10]. Different in vitro intestinal models with CDI, from simple batch to multistage continuous systems, have also been reported [11]. Batch cultures were mainly used to study CD interactions with fecal microbiota with the aim to test alternative treatments to antibiotics [12–15]. A three-stage intestinal model which may be more representative for colonic conditions, inoculated with pooled fecal microbiota from elderly donors was applied in a series of studies with different CD ribotypes and antibiotic treatments, as reviewed by Best et al. [11]. With this model CD spores were inoculated while the antibiotic clindamycin was used to induce CD spore germination [16–18]. However, monitoring of intestinal microbiota composition was limited to culture-based methods targeting a restricted range of gut bacteria, and no metabolic assessment was reported. To date, no study has reported in-depth analysis of CD colonization ability in in vitro continuous fermentation models using molecular methods and next generation sequencing to describe microbiota effects.

We have recently developed a new PolyFermS continuous model platform inoculated with immobilized fecal microbiota that mimics different sections of the elderly colon [19]. We showed that the models closely reproduce the gut microbiota composition, density and activity of the fecal donor. Furthermore, the PolyFermS platform allows simultaneous testing of several treatments compared to a control in parallel reactors inoculated with the same fecal microbiota generated in the upstream inoculum reactor (IR) seeded with fecal beads inoculated with single donor microbiota [20, 21]. The aim of the present study was to investigate colonization of CD vegetative cells and spores (PCR ribotype 001 and 012) in the new PolyFermS model of elderly colonic fermentation. Further, the effects of two antibiotics, ceftriaxone and metronidazole, were tested on the composition (qPCR, 16S pyrosequencing) and activity (HPLC) of the gut microbiota. CD growth and toxin production were analyzed with specific qPCR and Vero cell assays, respectively.

## Results

### Continuous fermentation models mimicking the elderly colon

**Fig. 1:**
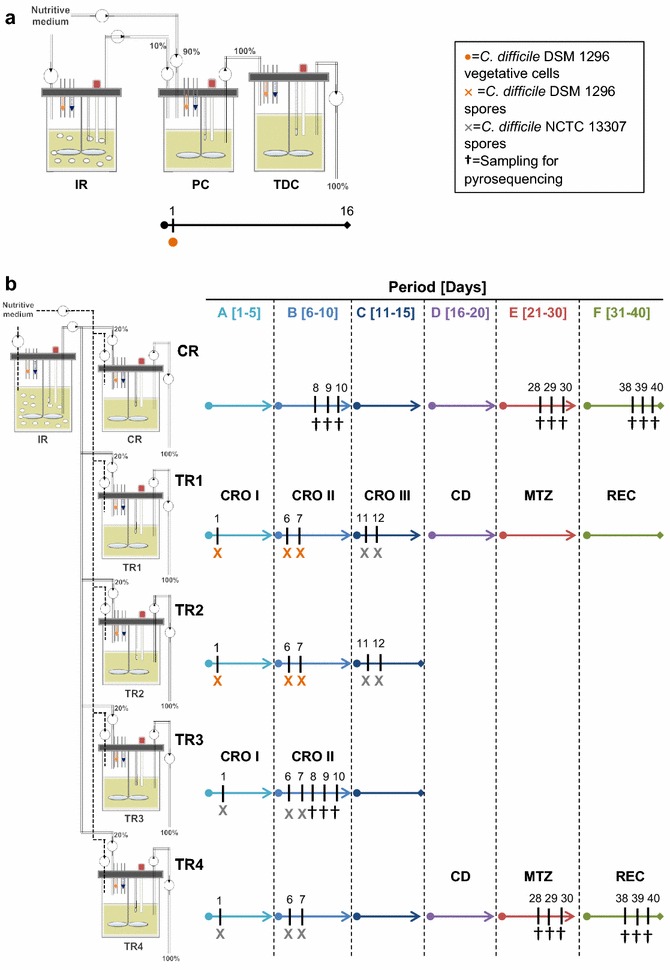
Experimental design of the two continuous fermentation models. **a** *Model 1* vegetative CD cells of strain DSM 1296 were added to PC on day 1 and growth was observed in PC and TDC during 16 days. **b** *Model 2* CR and TRs were fed with 100% effluent from IR during period A; and with fresh fermentation medium during periods B–F. Addition of CD spores (10^7^ cfu) of strain 1296 or 13307 was performed in TRs as indicated. IR, inoculum reactor; CR, control reactor; TR, test reactor. CRO I, 75 mg L^−1^ ceftriaxone addition twice daily; CRO II + III, 150 mg L^−1^ ceftriaxone addition once daily; CD, *C. difficile*; MTZ, metronidazole; REC, recovery

Testing of CD colonization and antibiotic treatments was performed in PolyFermS continuous fermentation models inoculated with fecal beads immobilizing elderly gut microbiota, and operated with conditions mimicking the elderly proximal (PC) and transverse-distal colon (TDC) [19]. Models 1 and 2 were composed of an inoculum reactor (IR) seeded with 30% (v/v) fecal beads, operated with proximal colon conditions, and used to inoculate (10% (v/v)) different second-stage reactors mounted in parallel. Mounted in series with IR, model 1 had two parallel sets of 2-stage reactors mimicking the proximal and distal colon whereas only one set was used to test CD colonization in this study. Model 2 was designed with five reactors mounted in parallel (one control CR and four treatment reactors TR1-4), and mimicking conditions of the transverse-distal colon regions (Fig. 1a, b). Investigation of the model microbiota using qPCR, pyrosequencing and HPLC revealed high diversity similar to the fecal donor’s microbiota, stable metabolic activity over long time operation periods of up to 76 days, and good reproducibility of control and test reactors inoculated with the same microbiota generated in the immobilized cell reactor (IR) within a PolyFermS model [19].

### Colonization of CD vegetative cells

In a preliminary test colonization of CD vegetative cells of strain DSM 1296, a clinical isolate of PCR ribotype 001, was evaluated in the continuous fermentation model 1 seeded with fecal beads of a 72-year old female donor. Vegetative cells of strain 1296 were spiked in PC (log_10_ 9.8 cfu in 250 mL), and CD colonization was monitored by qPCR using a specific primer during 16 days continuous fermentation (Table 1). After 6 h the gene copy (GC) number of CD was 7.4 and 7.3 log_10_ GC mL^−1^ in PC and TDC reactors, respectively. In both reactors a wash-out was observed until day 3, when CD numbers were below the detection limit of the qPCR test (log_10_ 4.4 GC mL^−1^). However, CD was detected again after day 9 of continuous culture in TDC, reaching high copy numbers of log_10_ 7.8 mL^−1^ after day 16. Since CD remained under detection limit in PC, fermentation model 2 was designed to target CD colonization in TDC conditions.

**Table 1:** Gene copy (GC) number of CD strain 1296 in proximal (PC) and transverse-distal (TDC) colon reactors of model 1

Days post CD instillation	Reactors	
	PC (GC mL^−1^)	TDC (GC mL^−1^)
0.25	7.4	7.3
1	5.7	6.2
2	4.4	5.1
3	ND	ND
4	ND	ND
5	ND	ND
6	ND	ND
7	ND	ND
8	ND	ND
9	ND	6.0
10	ND	6.0
11	ND	6.4
12	ND	6.9
13	ND	7.1
14	ND	7.5
15	ND	7.8
16	ND	7.8

Data are mean log_10_ 16S rRNA GC mL^−1^ fermentation effluent from 6 h after CD vegetative cells inoculation (log_10_ 9.8 cfu in 250 mL volume PC reactor) and during the following 16 days. Samples were analyzed in duplicate. The detection limit of qPCR of CD was log_10_ 4.4 GC mL^−1^

PC, proximal colon; TDC, transverse-distal colon; CD, *C. difficile*; ND, not detected

### Effect of ceftriaxone on gut microbiota composition, diversity and metabolites

**Fig. 2:**
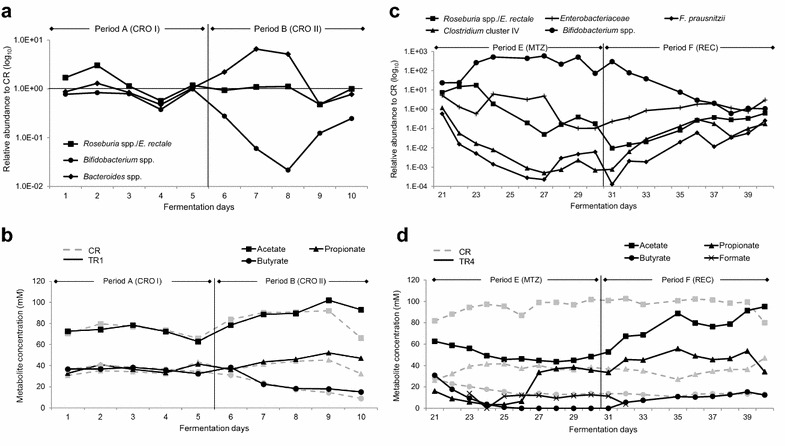
Effect of antibiotic treatment on gut microbial groups and metabolites. **a** Relative abundance of selected microbial groups tested with qPCR in CRO treated reactor TR1 relative to CR during period A and B. **b** Daily mean metabolites concentrations in TR1 and CR during period A and B assessed with HPLC. **c** Relative abundance of selected microbial groups tested with qPCR in MTZ treated reactor TR4 relative to CR during period E and F. **d** Daily mean metabolites concentrations in CR and MTZ treated reactor TR4 during period E and F. *CRO* ceftriaxone, *MTZ* metronidazole, *REC* recovery

**Fig. 3:**
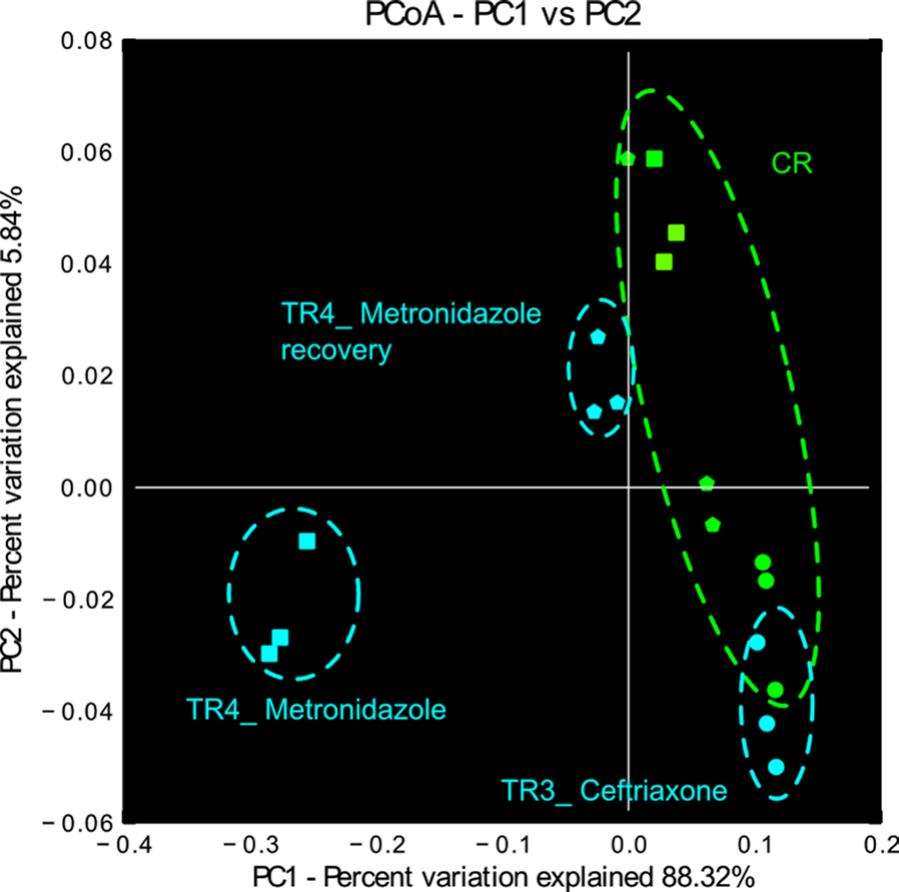
PCoA analysis during period B, E and F based on weighted UniFrac distance matrix. The three last days of period B (*circles*), period E (*squares*) and period F (*pentagons*) are presented for TR3 (period B) and TR4 (period E and F) and compared to the same CR period

Treatment with ceftriaxone (150 mg L^−1^ per day) was investigated in TDC conditions in model 2 inoculated with fecal beads containing the microbiota from a 78-year old woman (Fig. 1b). During the first period A CR and TRs (TDC conditions) were connected to IR and continuously fed with 100% effluent from IR mimicking PC conditions. No significant differences in bacterial group concentrations in effluent samples of antibiotic-treated reactors and CR were observed using qPCR (period A, Table 2; Fig. 2a). We therefore assumed that continuous supply of inoculum effluent from IR (100% v/v) may have masked the effect of the antibiotic on the gut microbiota. Consequently, for the next treatment period with ceftriaxone (period B) CR and TRs were disconnected from IR and were fed with fresh fermentation medium only. A significant decrease of 1.5 log_10_ in *Bifidobacterium* spp. was observed in both TR1 and TR3 upon ceftriaxone treatment compared to CR. Small but significant differences were observed for the copy numbers of *Clostridium* cluster IV (significant for TR3 and TR4) and *Roseburia* spp. (significant for TR3) that were lower in TRs relative to CR (period B, Table 2). The microbial composition and diversity of effluent samples of TR3 was determined with pyrosequencing and compared to CR during the last 3 days of the second ceftriaxone treatment (period B). A similar composition was measured at the genus level in TR3 and CR (Additional file 1), with overlapping clusters based on UniFrac distances (Fig. 3). The average Shannon diversity index measured in effluent samples of the three last days of period B in TR3 and CR were similar, with 3.9 ± 0.2 and 3.8 ± 0.2, respectively.

**Table 2:** Gene copy (GC) number of bacterial groups in TDC reactors of model 2 during antibiotic treatment [A, B and E] and recovery [F] periods

	Total 16S rRNA gene	*Bacteroides* spp.	*Entero*-*bacteriaceae*	*Lactobacillus* spp.	*Bifidobacterium* spp.	*F. prausnitzii*	*Clostridium* cluster IV	*Roseburia* spp./*E. rectale*
Period A (CRO I)								
CR	11.3 ± 0.2	10.3 ± 0.1	9.8 ± 0.2	7.3 ± 0.3	8.4 ± 0.3	9.0 ± 0.1	9.8 ± 0.2	7.4 ± 0.1
TR1	11.2 ± 0.2	10.1 ± 0.1	9.7 ± 0.1	7.3 ± 0.2	8.3 ± 0.4	8.9 ± 0.1	9.7 ± 0.2	7.4 ± 0.1
TR3	11.4 ± 0.07	10.1 ± 0.1	9.8 ± 0.3	7.5 ± 0.1	8.2 ± 0.1	8.9 ± 0.2	9.5 ± 0.5	7.4 ± 0.1
Period B (CRO II)								
CR	11.3 ± 0.4	10.3 ± 0.5	9.6 ± 0.3	8.3 ± 0.3	8.2 ± 0.2	8.9 ± 0.3	9.1 ± 0.2	6.5 ± 0.1
TR1	11.3 ± 0.04	10.6 ± 0.3	9.7 ± 0.2	8.3 ± 0.1	6.7 ± 0.2*	8.6 ± 0.4	8.8 ± 0.1*	6.0 ± 0.4
TR3	11.4 ± 0.1	10.5 ± 0.1	9.5 ± 0.1	8.5 ± 0.2	6.7 ± 0.3*	8.6 ± 0.5	8.8 ± 0.1*	6.1 ± 0.2*
Period E (MTZ)								
CR	10.9 ± 0.2	9.6 ± 0.2	10.4 ± 0.2	7.6 ± 0.2	7.1 ± 0.3	9.1 ± 0.1	9.7 ± 0.1	5.8 ± 0.1
TR1	11.2 ± 0.2	9.5 ± 0.2	9.4 ± 0.5*	7.7 ± 0.3	9.5 ± 0.2*	5.7 ± 0.1*	5.9 ± 0.2*	5.1 ± 0.2*
TR4	11.1 ± 0.1	9.7 ± 0.2	9.3 ± 0.3*	8.1 ± 0.3	9.4 ± 0.3*	6.7 ± 0.1*	6.7 ± 0.2*	5.0 ± 0.2*
Period F (REC)								
CR	11.3 ± 0.2	10.2 ± 0.1	10.1 ± 0.4	7.2 ± 0.5	7.1 ± 0.2	8.3 ± 0.2	9.7 ± 0.2	6.4 ± 0.1
TR1	11.7 ± 0.3	10.3 ± 0.1	10.1 ± 0.3	7.4 ± 0.4	7.6 ± 0.3*	8.2 ± 0.3	8.5 ± 0.03*	6.3 ± 0.1
TR4	11.3 ± 0.1	10.1 ± 0.2	10.3 ± 0.4	7.4 ± 0.1	7.1 ± 0.1	7.2 ± 0.2*	8.6 ± 0.2*	5.5 ± 0.02*

Data are mean log_10_ GC mL^−1^ fermentation effluent ± SD of the last 3 days of the treatment periods; samples were analyzed in duplicates

Means with an asterisk differ significantly from the control reactor within the same bacterial group (*p* < 0.05)

*CR* control reactor, *TR* test reactor, *CRO* ceftriaxone, *MTZ* metronidazole, *REC* recovery

Metabolite concentration profiles during fermentation periods A and B are illustrated for TR1 and compared to CR (Fig. 2b). Metabolite profiles in TR3 were similar to TR1 (Additional file 2). Mean metabolite concentrations measured during the three last days of each period for CR, TR1 and TR3 are presented in Table 3. Metabolite concentrations were similar in ceftriaxone treated reactors TR1 and TR3 and in CR during both periods A and B when reactors were connected and disconnected from IR, respectively. However, slight but significantly higher acetate concentration in TR3 period B were observed. Re-stabilization of the metabolites in CR after disconnection lasted approximately 5 days (Additional file 3).

**Table 3:** Metabolites concentration (mM) and ratios (%) measured by HPLC in model 2 during antibiotic treatment [A, B and E] and recovery [F] periods

	Acetate	Propionate	Butyrate	Formate	Total metabolites	SCFA Ratios (%)		
						Acetate	Propionate	Butyrate
Period A (CRO I)								
CR	75.7 ± 4.8	33.4 ± 2.5	38.4 ± 2.2	ND	147.5 ± 5.8	51.3	22.6	26.0
TR1	75.0 ± 3.0	36.7 ± 4.0	37.4 ± 0.9	ND	148.4 ± 6.4	20.5	24.7	24.7
TR3	76.5 ± 4.1	33.5 ± 2.0	35.8 ± 1.3	ND	145.8 ± 4.7	52.5	23.0	24.6
Period B (CRO II)								
CR	91.2 ± 0.9	43.6 ± 2.2	18.4 ± 4.6	ND	153.2 ± 5.2	59.5	28.5	12.0
TR1	93.5 ± 7.4	47.2 ± 4.3	19.5 ± 2.6	ND	160.2 ± 8.9	58.4	29.5	12.2
TR3	97.0 ± 5.1*	45.6 ± 4.3	19.6 ± 2.1	ND	162.2 ± 7.0	97	45.6	19.6
Period E (MTZ)								
CR	99.2 ± 2.4	37.0 ± 1.2	13.3 ± 0.5	ND	149.5 ± 2.7	66.4	24.7	8.9
TR1	44.1 ± 0.7*	35.9 ± 1.8	ND	10.6 ± 1.0	90.6 ± 2.2*	55.1	44.9	0
TR4	45.6 ± 2.5*	37.0 ± 1.3	ND	11.3 ± 1.6	93.9 ± 3.2*	55.2	44.8	0
Period F (REC)								
CR	92.6 ± 10.9	39.9 ± 6.2	13.4 ± 0.2	ND	145.9 ± 12.5	63.5	27.3	9.2
TR1	83.7 ± 0.4	43.8 ± 2.4	13.4 ± 0.7	ND	140.9 ± 2.5	59.4	31.1	9.5
TR4	88.4 ± 8.6	44.8 ± 9.8	13.5 ± 1.6	ND	146.7 ± 13.1	60.3	30.5	9.2

Data are mean ± SD of the three last days of treatment periods; samples were analyzed in duplicates

Means with an asterisk (*) differ significantly from the control reactor within the same metabolite (*p* < 0.05)

*CR* control reactor, *TR* test reactor, *SCFA* short-chain fatty acids, *CRO* ceftriaxone, *MTZ* metronidazole, *REC* recovery, *ND* not detected

### Colonization of CD spores and susceptibility to ceftriaxone

**Fig. 4:**
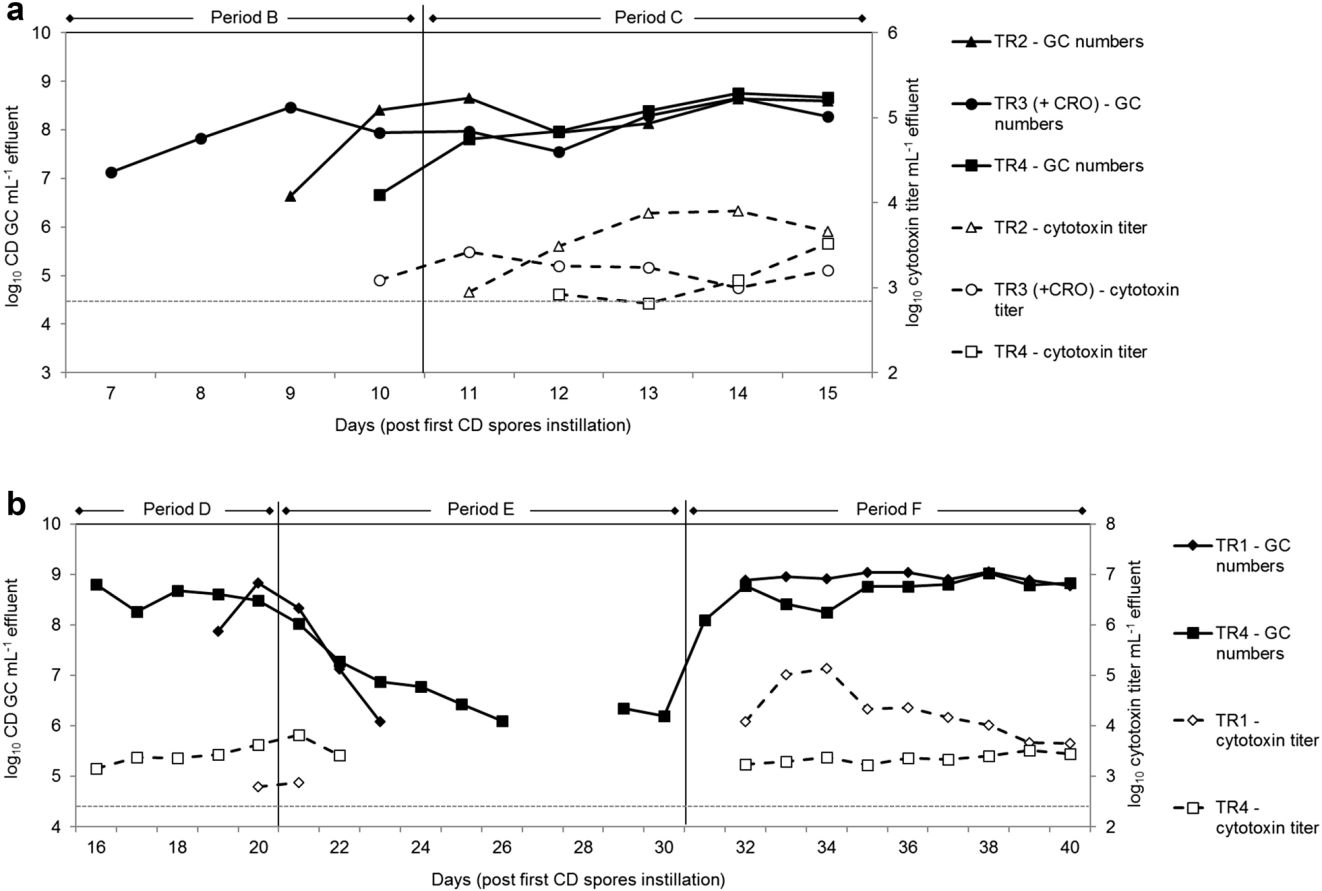
CD gene copy (GC) numbers and cytotoxin titers in TRs of model 2. **a** Period B and C in TR2-4. CD GC numbers and toxin titers are shown starting from first detection of CD growth in TR3 on day 7. TR1 was treated with CRO during period B and C but no CD growth was detected in this reactor during these periods. **b** Period D-F in TR1 and TR4. CD colonization was observed during period D. On day 21 metronidazole treatment [E] was performed in TR1 and TR4 for 10 days and followed by a recovery period [F] of 10 days. CD GC numbers and cytotoxin titer were determined by qPCR and Vero cell assays, respectively. (—) CD detection limit of 4.4 GC mL^−1^. CRO, ceftriaxone; CD, *Clostridium difficile*

The effect of ceftriaxone treatment on spore outgrowth and colonization of CD strains 1296 and 13307 was investigated in TRs of model 2 mimicking TDC conditions (Fig. 1). In fermentation period A (with CR and TRs connected to IR) CD spores (10^7^ cfu) were spiked in TR1 and TR2 (strain 1296), and in TR3 and TR4 (strain 13307). Treatment with ceftriaxone (75 mg L^−1^, twice daily) was performed simultaneously in TR1 and TR3 to induce spore germination. Unexpectedly, CD growth was not detected in any of the test reactors after 5 days. In fermentation period B (with CR and TRs disconnected from IR) a second inoculation of CD spores (10^7^ cfu), similar to period A, was applied on two consecutive days. Ceftriaxone treatment (150 mg L^−1^, once daily) was also applied in TR1 and TR3 for 5 days. CD strain 13307 growth was detected after 2 and 5 days in TR3 with and TR4 without antibiotic, respectively. CD strain 1296 was detected after 4 days in TR2 where no antibiotic was added (Fig. 4a).

During period C, CD spores (10^7^ cfu) of strain 13307 were instilled twice in TR1 (with ceftriaxone) where no growth of CD strain 1296 was detected during period B after 5 days, and in TR2 (without ceftriaxone). No effects were measured for the additional spore inoculation in TR2, while in TR1 CD growth was not detected after 5 days, but occurred on day 4 of period D when ceftriaxone was removed. CD GC numbers of log_10_ 8.8 mL^−1^ were measured at the end of period D (Fig. 4b).

Similar high CD numbers of 8.3–8.7 log_10_ GC mL^−1^ were determined in TR2, TR3 and TR4 15 days after the first inoculation of spores corresponding to the end of period C (Fig. 4a). Cytotoxin activity in TR2–TR4 was first detected shortly after CD cell growth, and ranged between 2.9 and 3.9 log_10_ cytotoxin titer mL^−1^, with highest cytoxin titers measured in TR2.

### Effect of metronidazole on gut microbiota composition, diversity and metabolites

**Fig. 5:**
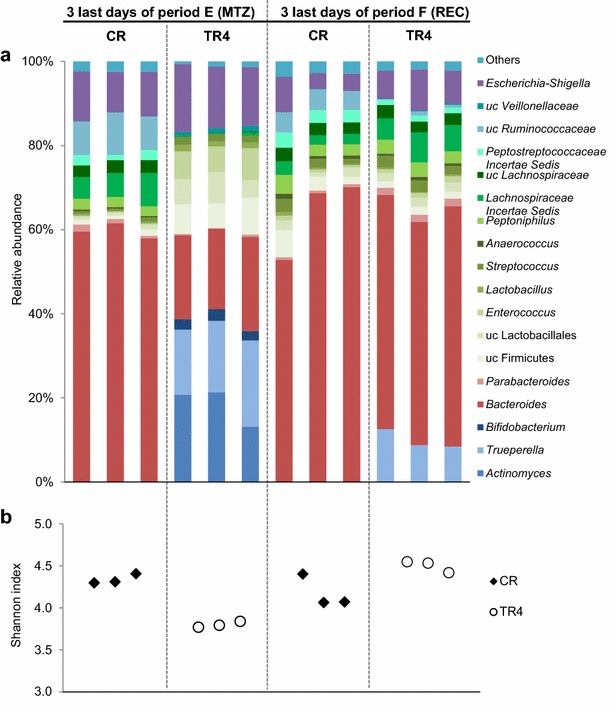
Effect of metronidazole on microbial composition and diversity measured by 454-pyrosequencing. The microbiota profile in reactor effluents of CR and TR4 during the last 3 days of period E and F was analyzed by 454-pyrosequencing of the V5–V6 hypervariable regions of the 16S rRNA gene. **a** Relative abundance at genus level. Values <1% are summarized in the group “others”. **b** Diversity measured by the Shannon diversity index. A higher Shannon diversity index reflects a more diverse community (in abundance and evenness). *MTZ* metronidazole, *REC* recovery, *uc* unclassified

The effect of metronidazole (333 mg L^−1^ added twice daily) on gut microbiota composition and metabolites in TR1 and TR4 was investigated in period E. The mean concentrations of bacterial groups analyzed with qPCR and metabolites calculated over the last 3 days of the period and compared with CR are presented in Tables 2 and 3. The relative abundance of selected bacterial groups analyzed with qPCR and the metabolite concentrations were determined during the entire fermentation period, and illustrated for TR4 and CR (Fig. 2c, d). The metabolic profile in TR1 was similar to TR4 (Additional file 4). Microbial diversity was additionally assessed with pyrosequencing during the last 3 days in CR and TR4 (Figs. 3, 5). Metronidazole considerably affected the microbial composition. A decrease in copy numbers of *Enterobacteriaceae*, *F. prausnitzii*, *Clostridium* cluster IV and *Roseburia* spp. of up to 3.8 log_10_ GC mL^−1^ was measured in TR1 and TR4 during the last 3 days of metronidazole treatment compared to CR (period E, Table 2). In contrast, an increase of *Bifidobacterium* spp. (2.4 and 2.3 log_10_ GC mL^−1^ in TR1 and TR4, respectively) was measured during metronidazole treatment compared to CR. Pyrosequencing of TR4 effluent samples revealed a profoundly altered microbial profile compared to CR. At the phylum level Bacteroidetes decreased from average values of 61% in CR to 21% in TR4 and Actinobacteria increased from less than 1% in CR to 39% in TR4, while Firmicutes and Proteobacteria exhibited less pronounced changes within 5% (Additional file 5). At the genus level, high relative abundance of up to 21% was observed in TR4 for *Trueperella*, *Actinomyces* and *Enterococcus* while these genera were at low relative abundance of less than 1% or undetected in CR (Fig. 5). The mean Shannon diversity index measured during the last 3 days of metronidazole treatment was lower in TR4 (3.8 ± 0.03) compared to CR (4.3 ± 0.1; Fig. 4). In addition, PCoA analysis showed a clear separation between samples from TR4 and CR during metronidazole period (Fig. 5).

Similarly, metronidazole also largely impacted gut microbiota metabolic activity, with a 50% decrease of acetate concentrations in TR1 and TR4 relative to CR, complete inhibition of butyrate production, and significant accumulation of formate (Table 3; Fig. 2d). Propionate concentrations decreased from 25 mM to approximately 4 mM in TR4 during the first 4 days of metronidazole treatment, but sharply increased between days 6 and 7 to reach stable concentrations of 37.0 ± 1.3 mM during the last 4 days.

### Growth and toxin production of CD during metronidazole treatment

During a 10-day treatment of metronidazole (period E) in TR1 and TR4 initially colonized with high CD numbers (log_10_ 8.8 and 8.5 GC mL^−1^, respectively), a rapid decrease of CD gene copy number was observed after only 1 day, reaching the detection limit of the qPCR test after 3 and 6 days in TR1 and TR4, respectively (Fig. 4b). The toxin production steadily decreased from 2.8 and 3.6 log_10_ cytotoxin titer mL^−1^ to non-detectable levels following 1 and 2 days of metronidazole treatment in TR1 and TR4, respectively. Unexpectedly CD was detected again at a concentration of log_10_ 6.3 GC mL^−1^ in TR4 on day 9 of antibiotic treatment during period E.

### Recovery of gut microbiota and CD after metronidazole treatment

The microbiota composition and metabolic activity of TR1 and TR4 was monitored over 10 days during period F after cessation of metronidazole treatment of period E. All bacterial populations analyzed with qPCR showed a recovery trend towards CR composition (Fig. 2c; Table 2). However, the populations of *F. prausnitzii* and *Clostridium* cluster IV were significantly lower in TRs compared to CR, while *Bifidobacterium* spp. remained higher in TR1 relative to CR at the end of period F, suggesting that the microbiota recovery was incomplete after 10 days (Table 2). Pyrosequencing data at the phylum level showed similar relative abundance for Bacteroidetes in TR4 (57%) compared to CR (64%), but Actinobacteria levels remained higher in TR4 than in CR (10% versus below 1%, Additional file 5). Most genera that increased during metronidazole treatment decreased or became undetectable during the last 3 days of the recovery period (Fig. 5). The Shannon diversity index determined at the end of the recovery period was similar in TR4 (4.5 ± 0.07) compared to CR (4.2 ± 0.2) (Fig. 5). In addition, PCoA analysis revealed that microbial composition of the last 3 days of the recovery period in TR4 clustered close to samples from CR of the same treatment period (Fig. 3).

Metabolite concentrations in TR1 and TR4 measured during the last 3 days of period F were not significantly different from that in CR (Table 3; Fig. 2d; Additional file 4: Figure S4).

The CD growth and toxin production after cessation of metronidazole treatment was monitored for 10 days (period F, Fig. 4b). After only 2 days, CD was tested at high numbers of approximately log_10_ 9 GC mL^−1^ in both TR1 and TR4 and toxin production was detected. The toxin titer in TR1 increased until day 5 to reach 5 log_10_ cytotoxin titer mL^−1^, and steadily decreased thereafter to 3.7 log_10_ cytotoxin titer mL^−1^ after 10 days. In contrast toxin production in TR4 remained constant from day 2 to 10 (mean value of 3.34 ± 0.09).

## Discussion

In vitro intestinal fermentation models are useful tools to investigate colonization of enteropathogens, such as Salmonella, and interactions with the human gut microbiota, independent of the effects of the host [22–24]. The human colon can be approximated to a continuous fermenter, divided in regions (ascending, transverse and descending colonic) that are different in metabolic activity and microbial composition. A major advancement of in vitro gut fermentation models was the development of multistage continuous fermentation models which enable the simulation of horizontal colon processes [25]. Investigation of CD colonization in intestinal models has been primarily performed with three-stage colonic models, inoculated with a liquid fecal suspension from pooled donors, using cultivation methods for microbiota analysis [16, 18].

In a recent study we investigated novel in vitro continuous colonic fermentation models inoculated with immobilized fecal microbiota from single elderly donors and characterized in detail microbiota composition, diversity and activity [19]. Immobilization of fresh gut microbiota reproduced both the planktonic (free-cell) and sessile (biofilm-associated) states of bacterial populations in the colon and yielded self-contained continuous fermentation system of high cell density, diversity and population stability, similar to what is observed in the human GI tract [9]. In this study, we used two fermentation models to investigate the colonization of CD vegetative cells or spores, and the response of the microbiota to antibiotics known to induce or used to treat CDI.

It is suggested that the small intestine is the site of CD spores germination into the metabolically active vegetative cells [26, 27], which then proliferate in the large intestine and cause symptoms typical for CDI [5, 28]. Using PolyFermS models, we showed that CD vegetative cells or spores only colonize in TDC reactors after a time period of 7–10 days. Our data are in agreement with previous studies that reported an absence of CD colonization added as spores in the proximal colon section of a three stage colonic fermentation model [29, 30], which may be explained by pH inhibition effect [31].

The CD concentration determined in TDC reactors of our models (from 6.0 to 8.9 GC mL^−1^) is within the range of in vivo qPCR data for feces of antibiotic-associated diarrhea patients (aged 3–89 years; from 5.6 to 11.2 cell equivalents log_10_ g^−1^ [32]). Growth of CD in gut models upon inoculation of vegetative cells was only reported for batch fermentations, yielding cell counts up to 8 log_10_ cfu mL^−1^ after 24 h [12, 13]. Although we used a high inoculum of vegetative cells (log_10_ 9.8 cfu) in model 1, CD cells were washed-out and remained undetected for a period of 6 days after which they were shown to colonize in TDC reactors.

The PolyFermS design of model 2 was therefore selected to mimic TDC conditions for CD colonization. We investigated the colonization of two PCR ribotypes of CD spores and the effects of two antibiotics, ceftriaxone and metronidazole, that are known to respectively promote and treat infection. CD remained undetected in all TRs for 5 days after which a repeated inoculation of spores was performed. This result suggests that either the spores were washed out of the system, or that they were able to grow, but remained below the detection limit of the qPCR method (4.4 GC mL^−1^). A quiescent state of CD spores for a period of at least 7 days was previously reported in a three-stage model, and was tentatively explained by the absence of antibiotic treatment [29, 30]. Our data demonstrate that application of antibiotic may not be required for CD spore germination and colonization of an in vitro continuous colonic fermentation model mimicking TDC conditions of an elderly microbiota. This is in contrast to in vivo data in humans and animal models suggesting that CD infection is promoted by a disturbed microbiota mainly due to antibiotic treatment [6, 33]. This discrepancy between in vitro and in vivo CD colonization may be a consequence of host-specific mechanisms, including the epithelial cell layer and immune responses, both of which are lacking in in vitro models. Lawley et al. [34] showed that CD can asymptomatically colonize mice in the absence of antibiotic treatment while antibiotics were necessary for CD to proliferate and sporulate. The study suggested that toll-like receptor signaling may protect against virulent CD overgrowth.

Ceftriaxone is a broad-spectrum antibiotic that is associated with a high risk of developing diarrhea due to CD colonization [35, 36]. Pletz et al. [37] reported that in the fecal microbiota of ten healthy adults, ceftriaxone treatment (2 g administered once daily for 7 days) induced a marked decrease in lactobacilli, bifidobacteria, clostridia and *Bacteroides*, with an attendant increase in enterococci. In the present study, we observed only minor effects of ceftriaxone on the gut microbiota when added at 150 mg L^−1^ to mimic ceftriaxone levels found in feces of volunteers from the study mentioned above [37] on the 4th day of the treatment. *Bifidobacterium* spp. was most affected, but diversity and metabolic activity was not changed compared to the control reactor. The detrimental effect of ceftriaxone on *Bifidobacterium* spp. was also observed in the human gut model of Baines [29] when the same antibiotic concentration was used to induce CD spore germination. In contrast to our study, Baines only used plating for gut microbiota enumeration (total facultative anaerobes, total anaerobes (facultative + obligate), lactose-fermenting *Enterobacteriaceae*, enterococci, lactobacilli, bifidobacteria, total *Clostridium* spp. and *Bacteroides fragilis* group), and did not test diversity and metabolic effects.

Metronidazole is used in the treatment of anaerobic infections [38], including cases of mild to moderate CDI [39]. We measured an extreme shift of the gut microbiota composition during metronidazole treatment, with a very large increase of Actinobacteria at the expense of Bacteroidetes and sharp inhibition of butyrate production. A detrimental effect of metronidazole on Bacteroidetes was also observed in a previous single-stage human distal colon model which was used to investigate the activity of a bacteriocin versus antibiotics against CD, however, metronidazole mainly promoted growth of bacteria belonging to the phylum Proteobacteria [13]. In previous in vitro studies which used concentrations varying greatly between 9.3 mg L^−1^ every 8 h [17] to 330 mg L^−1^ per day [14] no effect on CD counts and toxin production was observed with the low antibiotic level [17]. However, CD eradication was reported for the higher concentration in a batch fermentation which contained a total anaerobes concentration of approximately log_10_ 10.3 cfu mL^−1^, estimated with plate counts [14]. In our model we chose to apply a higher antibiotic concentration (333 mg L^−1^, twice daily) to account for the high total cell numbers tested in TDC (in the range of log_10_ 11.1–11.2 GC mL^−1^ [19]). Furthermore, we considered that the concentration of metronidazole entering the colon is higher than that tested in feces since the gut bacteria metabolize the drug [40]. With our conditions, we observed a strong reduction of CD numbers while toxin production decreased below the detection limits.

An important issue to consider with treatment of CDI is the high recurrence rate after antibiotic treatment, typically around 27% after metronidazole treatment [41]. Our data consistently demonstrate that a fast recurrence of CD occurred only 2 days after cessation of the metronidazole treatment, also supporting the applied metronidazole dosage. Gut bacterial groups that were mainly affected by metronidazole (*Clostridium* cluster IV, *F. prausnitzii* and *Roseburia* spp.) showed only partial recovery to control levels after 10 days. To our knowledge, this is the first report CD recovery after metronidazole treatment in a gut fermentation model.

## Conclusions

In conclusion, our study provides evidence to support the use of continuous intestinal models for the in vitro investigation of CD colonization dynamics and antibiotic treatments on elderly gut microbiota independent of host factors. We demonstrated colonization of vegetative cells and spores of CD in the gut microbiota under conditions of the transverse-distal colon without requirement of an antibiotic treatment. Treatment with metronidazole induced sharp deleterious effects on the gut microbiota composition and activity at the tested dosage. However, even when applied at high dosage, metronidazole only induced a temporary inhibition of CD followed by a rapid recurrence after cessation of treatment. Immobilization of fecal microbiota in gel beads allowed the investigation of a high bacterial cell density similar to in vivo gut microbiota. The PolyFermS models proved to be well-suited for direct comparison of treatment effect to control conditions in reactors inoculated with the same microbiota and mimicking TDC conditions. However, our data suggest that strong host effects are involved with CD protection in the gut as suggested by colonization occurring in the absence of antibiotics. Consequently, in vitro models lacking host factors may only provide partial understanding of the CD colonization mechanisms involved with CDI. Nevertheless, in vitro modeling is an important component, especially when combined with in vivo investigation of multi-scale strategy for gut microbiota investigations and for testing new antimicrobial treatments for their activity against CD [10].

## Methods

### *Clostridium difficile* strains

*Clostridium difficile* DSM 1296 (PCR ribotype 001) and *C. difficile* NCTC 13307 (PCR ribotype 012) were purchased from the Deutsche Sammlung von Mikroorganismen und Zellkulturen (DSMZ, Braunschweig, Germany) and the National Collection of Type Cultures (NCTC, Salisbury, UK), respectively. Vegetative cells of CD DSM 1296 and NCTC 13307 were cultured from glycerol stocks (33%, −80 °C) and routinely grown in BHI supplemented with 0.05% (w/v) l-cysteine (BHIS) in anaerobic Hungate tubes. Vegetative CD cells for inoculation of reactors were grown in serum flasks containing fermentation medium at 37 °C for 15 h. The medium composition was based on that described by MacFarlane et al. [25] for simulation of adult ileal chyme, and prepared according to Fehlbaum et al. [19]. Anaerobic conditions in serum flasks were achieved by flushing the headspace with N_2_ and CO_2_ at 3:1 ratio. Spores of CD DSM 1296 and NCTC 13307 were prepared according to Sorg and Dineen [42]. Briefly, several large petri dishes were spread with 150 µL of an overnight culture of CD and incubated in an anaerobic chamber at 37 °C for 10 days. Growth on the agar was collected by flooding the agar with sterile ice-cold water. The suspension of vegetative cells and spores was kept at 4 °C overnight in order to enhance the release of spores from mother cells. The suspension was subsequently centrifuged (14,000*g*, 1 min) and the pellet was washed several times with ice-cold water. Histodenz (Sigma-Aldrich) was used to separate the free spores from vegetative bacteria and cell debris. The spore pellet was washed several times with ice-cold water and finally resuspended in 200 µL sterile water. For spore enumeration a sample was plated on BHI agar containing 0.1% taurocholate (Sigma) and incubated for 48 h in an anaerobic chamber at 37 °C. Spore suspensions were adjusted to a concentration of 10^7^ cfu mL^−1^ using sterile water, and kept at 4 °C until further use.

### Antibiotics

Rocephin® (ceftriaxone sodium, >90% purity, Roche, Pharma AG, Reinach, Switzerland) was diluted in sterile ultra-pure water (1 g in 10 mL) and 340 µL of the solution was added once a day, or in two separate doses, to the reactors corresponding to a total added amount of 150 mg L^−1^. This concentration was adapted from Baines et al. [29] and is based on fecal ceftriaxone levels found in feces of volunteers with a mean concentration of 152 mg kg^−1^ on day four of treatment [37].

Crystalline metronidazole (Sigma-Aldrich, Buchs, Switzerland) was added twice daily directly to the reactors to achieve a final concentration of 333 mg L^−1^ with each addition. The treatment recommendations for metronidazole during CDI range between 500 and 750 mg three times per day [6, 43] and it was suggested that 6–15% of the antibiotic metabolites are extracted in feces [44]. We chose to apply a metronidazole concentration higher than tested in feces in vivo to account for absorption and possible degradation of the antibiotic during colonic transit (see discussion section).

### Fermentation medium

The fermentation medium was based on the composition described by MacFarlane et al. [25] for simulation of adult ileal chyme. Ingredients and preparation were described previously [19].

### Fecal inoculum and fermentation setup

The experimental setup of the two continuous in vitro fermentation models (PolyFermS models) is schematically illustrated in Fig. 1, and a detailed description of the experimental procedure was previously reported by Fehlbaum et al. [19]. Briefly, fresh fecal samples were obtained from two healthy women aged 72 (model 1) and 78 (model 2), who did not receive antibiotic treatment for at least three months prior to sample collection and who did not consume probiotics on a regular basis. The fecal microbiota was processed and immobilized in gellan-xanthan beads as described previously [19].

The two models were composed of a first inoculum reactor (IR) seeded with 30% (v/v) polysaccharide fecal beads, operated with conditions selected for the elder proximal colon (PC, mean retention time of 9 h, pH 5.7). In the preliminary setup (model 1) the IR was used to continuously inoculate a reactor (10% v/v IR effluent and 90% fresh medium) operated with PC conditions (retention time of 9 h, pH 5.7) that was connected in series to a second reactor mimicking transverse-distal colon conditions (TDC, retention time of 18 h, pH 6.8). For model 2 IR mimicking PC conditions was used to feed (100%) five reactors mounted in parallel [one control reactor (CR) and four test reactors (TR1-4)], and operated with TDC conditions (pH 6.8). During periods B to F, CR and TRs were disconnected from IR and were fed with fresh fermentation medium only to avoid masking of the effect of the antibiotic on the gut microbiota resulting from continuous supply of 100% effluent from IR (see “Results” section). The retention time of CR and TRs was set to 25 h, which is also in the range of in vivo measured retention times for elderly and to account for the overall fermentation taking place in one reactor compared to two in model 1 [19].

### Experimental design

Establishment and detailed characterization of the colonization and stability of the two continuous colonic fermentation models have previously been described [19].

Model 1 was used to test colonization of vegetative cells CD 1296 in PC and TDC conditions. CD cells (log_10_ 9.8 cfu) were spiked into PC (250 mL fermentation volume) after 18 days of model stabilization, indicated as day 1 (Fig. 1). Effluents of PC and TDC reactors were analyzed after 6 h and daily over a total period of 16 days.

Model 2 was used to test the germination of spores and colonization of vegetative cells of two CD ribotypes, with and without simultaneous treatment with antibiotics (ceftriaxone or metronidazole). Treatments were applied in TR1-4 operated at TDC conditions during six consecutive periods over a total fermentation time of 40 days (Fig. 1). CR served as control reactor during the entire experiment, with no treatment applied. During period A, CD spores (10^7^ cfu) were added once in TR1 and TR2 (strain 1296) and TR3 and TR4 (strain 13307) after 14 days of model stabilization (indicated as day 1). Rocephin® was supplied twice daily for 5 days in TR1 and TR3 at a concentration of 75 mg L^−1^ per injection in the fermentation volume (Period A). Since no effect of antibiotic was observed on microbiota composition and activity in TRs, we assumed that continuous feeding of effluent with microbiota grown at high concentration in IR masked the possible antibiotic effects. Therefore, for the subsequent tests (Period B–F), we chose to disconnect CR and TRs, all colonized by stable and similar microbiota from IR, and fed them 100% fresh fermentation medium. During period B, CD spores (10^7^ cfu, strain 1296 in TR1 and TR2, and strain 13307 in TR3 and TR4) were inoculated on two consecutive days (days 6 and 7) and ceftriaxone was added once daily for 5 days (150 mg L^−1^) to TR1 and TR3. During period C spores of strain 13307 were added to TR1 because no CD colonization was detected after application of spores of strain 1296 during period B. CD was allowed to colonize reactors TR1 and TR4 during another 5 days (period D) while TR2 and TR3 were used to test other treatments (data not shown). During period E, addition of metronidazole (333 mg L^−1^ twice daily) was tested in TR1 and TR4 during 10 days, followed by 10 days recovery (period F) without antibiotic addition.

### qPCR analysis

Genomic DNA was extracted from fermentation effluent samples (2 mL) using the FastDNA® SPIN Kit for Soil (MP Biomedicals, Illkirch, France) and a final elution volume of 100 µL. Total bacteria and specific bacterial groups prevalent in the gut were enumerated using previously described primers [19]. CD was quantified using primers (forward, 5′-TTG AGC GAT TTA CTT CGG TAA AGA-3′ and reverse, 5′-CCA TCC TGT ACT GGC TCA CCT-3′) and conditions described previously [45]. One µL of 10- or 100-fold diluted DNA was amplified in a total volume of 25 µL as described in Zihler et al. [23] using 2× SYBR Green PCR Master Mix (Applied Biosystems, Zug, Switzerland). Each reaction was run in duplicate on an ABI PRISM 7500-PCR sequence detection system (Applied Biosystems). For quantification, standard curves were produced by amplification of the DNA of the reference strain of the respective target group.

### HPLC analysis

Short chain fatty acids (SCFA; acetate, propionate, butyrate and valerate), lactate, formate and branched-chain fatty acids (BCFA; isobutyrate and isovalerate) in fermentation effluent samples from all reactors were determined daily by HPLC analysis in duplicate (Thermo Fisher Scientific Inc. Accela, Wohlen, Switzerland) [20]. Effluent supernatants (500 µL) were twofold diluted with sterile ultra-pure water and filtered directly into vials through a 0.45 µm nylon HPLC filter (Infochroma AG, Zug, Switzerland). The analysis was run at a flow rate of 0.4 mL min^−1^ using an Aminex HPX-87H column (Bio-Rad Laboratories AG, Reinach, Switzerland) and 10 mM H_2_SO_4_ as eluent.

### Microbiota profiling by 454 pyrosequencing

454-pyrosequencing analysis of total genomic DNA was carried out at DNAVision (Gosselies, Belgium). The V5–V6 hypervariable 16S rRNA region was amplified using specific primers 784F (5′-AGGATTAGATACCCTKGTA-3′) and 1061R (5′-CRRCACGAGCTGACGAC-3′) [46]. The forward primer contained the sequence of the Titanium A adaptor and a unique barcode sequence. Pyrosequencing was carried out using primer A on a 454 Life Sciences Genome Sequencer FLX instrument (Roche Applied Science, Vilvoorde, Belgium) following Titanium chemistry. The obtained data was analyzed using the open source software package Quantitative Insights Into Microbial Ecology (QIIME), v1.7 [47]. Briefly, raw sequencing reads were filtered based on selected quality criteria such as: (1) no mismatch with the primer sequences and barcode tags; (2) no ambiguous bases (Ns); (3) read-lengths not shorter than 200 base pairs (bp) or longer than 1000 bp; (4) the average quality score in a sliding window of 50 bp not to fall below 25; (5) excluding homopolymer runs higher than 6 nt. Sequences that passed quality filtering were clustered into OTUs at 97% identity level using usearch [48]. Representative sequences (the most abundant) for each OTU were aligned using PyNAST and taxonomically assigned using Greengenes v_13_08 database. These phylogenies were combined with absence/presence or abundance information for each OTU to calculate unweighted or weighted UniFrac distances, respectively using rarefaction of 7000 sequences per samples. Principal coordinates analysis (PCoA) was applied to the distance matrices for visualization. Alpha diversity (diversity within sample) was calculated using Shannon (evenness) indexes.

### Vero cell analysis

CD toxin production in reactor effluents of model 2 was estimated using Vero cell cytotoxicity assays as previously described [49, 50]. Briefly, 500 µL reactor effluent samples were centrifuged (10,000*g*, 10 min) and the supernatant was filtered through a 0.45 µm membrane filter (Infochroma AG, Zug, Switzerland) and subsequently serially diluted in peptone buffered saline (PBS) to 10^−7^. Twenty µL thereof was then mixed with 30 µL of cell media Dulbecco’s Modified Eagle’s medium (DMEM, Life Technologies) and added in duplicate to Vero cell culture monolayers prepared in 96-well microtiter plates. The cell culture toxin assay trays were incubated at 37 °C in 5% CO_2_ atmosphere and read after 48 h under an inverted microscope. A positive cytotoxin activity was indicated by cell rounding, with the end-point titer defined as the last dilution at which 50% rounded cells were measured. The action of CD cytotoxin in the samples was confirmed by neutralization with CD antitoxin (Alere Health BV, Tilburg, NL, USA).

### Statistical analysis

Statistical analysis of HPLC and qPCR data was performed using JMP 8.0 (SAS Institute Inc., Cary, NC, USA). HPLC and qPCR data are expressed as mean ± SD of the last 3 days of each treatment period. qPCR data were log_10_-transformed. qPCR and HPLC data among reactors were compared using the nonparametric Kruskal–Wallis test. *p* Values <0.05 were considered significant.

## Additional files

**Additional file 1.** Effect of ceftriaxone on the microbial composition measured by 454-pyrosequencing on genus level. The microbiota profile in effluents of CR and TR3 of model 2 during the three last days of period B was analyzed by 454-pyrosequencing of the V5-V6 hypervariable regions of the 16S rRNA gene. TR3 was treated with ceftriaxone (=CRO II) during the entire five days of period B. Values < 1% are summarized in the group “others”; uc, unclassified.

**Additional file 2.** Daily mean metabolites concentrations in ceftriaxone (=CRO) treated TR3 compared to CR during period A (CRO I) and B (CRO II).

**Additional file 3.** Daily mean SCFA concentrations in fermentation effluents of CR of model 2 measured by HPLC. From period B on CR was disconnected from continuous feed from IR; (♦) acetate, (■) propionate, (●) butyrate.

**Additional file 4.** Daily mean metabolites concentrations in metronidazole (=MTZ) treated TR1 compared to CR during period E and recovery period F (REC).

**Additional file 5.** Effect of metronidazole on the microbial composition measured by 454-pyrosequencing on the phylum and family level. The microbiota profile in reactor effluents of the last three days of period E and F was analyzed by 454-pyrosequencing of the V5-V6 hypervariable regions of the 16S rRNA gene. (A) Relative abundance at phylum level and (B) relative abundance at family level. Values < 1% are summarized in the group “others”. MTZ, metronidazole; REC, recovery; uc, unclassified.

## Abbreviations

CD: *Clostridium difficile*
CDI: *Clostridium difficile* infection
GC: gene copy
IR: inoculum reactor
PC: proximal colon
TDC: transverse-distal colon
CR: control reactor
TR: test reactor
CRO: ceftriaxone
MTZ: metronidazole
REC: recovery
SCFA: short chain fatty acids

## References

[1] Zucca M, Scutera S, Savoia D (2013). Novel avenues for *Clostridium difficile* infection drug discovery. Expert Opin Drug Discov.

[2] Rupnik M, Wilcox MH, Gerding DN (2009). *Clostridium difficile* infection: new developments in epidemiology and pathogenesis. Nat Rev Microbiol.

[3] Adlerberth I, Huang H, Lindberg E, Aberg N, Hesselmar B, Saalman R, Nord CE, Wold AE, Weintraub A (2014). Toxin-producing *Clostridium difficile* strains as long-term gut colonizers in healthy infants. J Clin Microbiol.

[4] Kamada N, Chen GY, Inohara N, Nunez G (2013). Control of pathogens and pathobionts by the gut microbiota. Nat Immunol.

[5] Vedantam G, Clark A, Chu M, McQuade R, Mallozzi M, Viswanathan VK (2012). *Clostridium difficile* infection: toxins and non-toxin virulence factors, and their contributions to disease establishment and host response. Gut Microbes.

[6] Oldfield IEC, Oldfield IEC, Johnson DA (2014). Clinical update for the diagnosis and treatment of *Clostridium difficile* infection. World J Gastrointest Pharmacol Ther.

[7] Deneve C, Janoir C, Poilane I, Fantinato C, Collignon A (2009). New trends in *Clostridium difficile* virulence and pathogenesis. Int J Antimicrob Agents.

[8] Gough E, Shaikh H, Manges AR (2011). Systematic review of intestinal microbiota transplantation (fecal bacteriotherapy) for recurrent *Clostridium difficile* infection. Clin Inf Dis.

[9] Payne AN, Zihler A, Chassard C, Lacroix C (2012). Advances and perspectives in in vitro human gut fermentation modeling. Trends Biotechnol.

[10] Lacroix C, de Wouters T, Chassard C (2015). Integrated multi-scale strategies to investigate nutritional compounds and their effect on the gut microbiota. Curr Opin Biotechnol.

[11] Best EL, Freeman J, Wilcox MH (2012). Models for the study of *Clostridium difficile* infection. Gut Microbes.

[12] Hopkins MJ, Macfarlane GT (2003). Nondigestible oligosaccharides enhance bacterial colonization resistance against *Clostridium difficile* in vitro. Appl Environ Microbiol.

[13] Rea MC, Dobson A, O’Sullivan O, Crispie F, Fouhy F, Cotter PD, Shanahan F, Kiely B, Hill C, Ross RP (2011). Effect of broad- and narrow-spectrum antimicrobials on *Clostridium difficile* and microbial diversity in a model of the distal colon. Proc Natl Acad Sci USA.

[14] Meader E, Mayer MJ, Gasson MJ, Steverding D, Carding SR, Narbad A (2010). Bacteriophage treatment significantly reduces viable *Clostridium difficile* and prevents toxin production in an in vitro model system. Anaerobe.

[15] Tejero-Sarinena S, Barlow J, Costabile A, Gibson GR, Rowland I (2013). Antipathogenic activity of probiotics against *Salmonella* Typhimurium and *Clostridium difficile* in anaerobic batch culture systems: is it due to synergies in probiotic mixtures or the specificity of single strains?. Anaerobe.

[16] Baines SD, O’Connor R, Saxton K, Freeman J, Wilcox MH (2009). Activity of vancomycin against epidemic *Clostridium difficile* strains in a human gut model. J Antimicrob Chemother.

[17] Freeman J, Baines SD, Saxton K, Wilcox MH (2007). Effect of metronidazole on growth and toxin production by epidemic *Clostridium difficile* PCR ribotypes 001 and 027 in a human gut model. J Antimicrob Chemother.

[18] Freeman J, Baines SD, Jabes D, Wilcox MH (2005). Comparison of the efficacy of ramoplanin and vancomycin in both in vitro and in vivo models of clindamycin-induced *Clostridium difficile* infection. J Antimicrob Chemother.

[19] Fehlbaum S, Chassard C, Haug MC, Fourmestraux C, Derrien M, Lacroix C (2015). Design and investigation of PolyFermS in vitro continuous fermentation models inoculated with immobilized fecal microbiota mimicking the elderly colon. PLoS ONE.

[20] Tanner SA, Zihler Berner A, Rigozzi E, Grattepanche F, Chassard C, Lacroix C (2014). In vitro continuous fermentation model (PolyFermS) of the swine proximal colon for simultaneous testing on the same gut microbiota. PLoS ONE.

[21] Zihler Berner A, Fuentes S, Dostal A, Payne AN, Vazquez Gutierrez P, Chassard C, Grattepanche F, de Vos WM, Lacroix C (2013). Novel Polyfermentor intestinal model (PolyFermS) for controlled ecological studies: validation and effect of pH. PLoS ONE.

[22] Tanner SA, Chassard C, Zihler Berner A, Lacroix C (2014). Synergistic effects of *Bifidobacterium thermophilum* RBL67 and selected prebiotics on inhibition of *Salmonella* colonization in the swine proximal colon PolyFermS model. Gut Pathog.

[23] Zihler A, Gagnon M, Chassard C, Hegland A, Stevens MJ, Braegger CP, Lacroix C (2010). Unexpected consequences of administering bacteriocinogenic probiotic strains for *Salmonella* populations, revealed by an in vitro colonic model of the child gut. Microbiology.

[24] Le Blay G, Rytka J, Zihler A, Lacroix C (2009). New in vitro colonic fermentation model for *Salmonella* infection in the child gut. FEMS Microbiol Ecol.

[25] Macfarlane GT, Macfarlane S, Gibson GR (1998). Validation of a three-stage compound continuous culture system for investigating the effect of retention time on the ecology and metabolism of bacteria in the human colon. Microb Ecol.

[26] Carlson PE, Kaiser AM, McColm SA, Bauer JM, Young VB, Aronoff DM, Hanna PC (2015). Variation in germination of *Clostridium difficile* clinical isolates correlates to disease severity. Anaerobe.

[27] Koenigsknecht MJ, Theriot CM, Bergin IL, Schumacher CA, Schloss PD, Young VB (2014). Dynamics and establishment of *Clostridium difficile* infection in the murine gastrointestinal tract. Infect Immun.

[28] Paredes-Sabja D, Bond C, Carman RJ, Setlow P, Sarker MR (2008). Germination of spores of *Clostridium difficile* strains, including isolates from a hospital outbreak of *Clostridium difficile*-associated disease (CDAD). Microbiology.

[29] Baines SD, Noel AR, Huscroft GS, Todhunter SL, O’Connor R, Hobbs JK, Freeman J, Lovering AM, Wilcox MH (2011). Evaluation of linezolid for the treatment of *Clostridium difficile* infection caused by epidemic strains using an in vitro human gut model. J Antimicrob Chemother.

[30] Baines SD, Chilton CH, Crowther GS, Todhunter SL, Freeman J, Wilcox MH (2013). Evaluation of antimicrobial activity of ceftaroline against *Clostridium difficile* and propensity to induce *C. difficile* infection in an in vitro human gut model. J Antimicrob Chemother.

[31] Lawley TD, Walker AW (2013). Intestinal colonization resistance. Immunology.

[32] Naaber P, Stsepetova J, Smidt I, Ratsep M, Koljalg S, Loivukene K, Jaanimae L, Lohr IH, Natas OB, Truusalu K, Sepp E (2011). Quantification of *Clostridium difficile* in antibiotic-associated-diarrhea patients. J Clin Microbiol.

[33] Hutton ML, Mackin KE, Chakravorty A, Lyras D (2014). Small animal models for the study of *Clostridium difficile* disease pathogenesis. FEMS Microbiol Lett.

[34] Lawley TD, Clare S, Walker AW, Goulding D, Stabler RA, Croucher N, Mastroeni P, Scott P, Raisen C, Mottram L (2009). Antibiotic treatment of *Clostridium difficile* carrier mice triggers a supershedder state, spore-mediated transmission, and severe disease in immunocompromised hosts. Infect Immun.

[35] Bartlett JG, Gerding DN (2008). Clinical recognition and diagnosis of *Clostridium difficile* infection. Clin Infect Dis.

[36] Privitera G, Scarpellini P, Ortisi G, Nicastro G, Nicolin R, de Lalla F (1991). Prospective study of *Clostridium difficile* intestinal colonization and disease following single-dose antibiotic prophylaxis in surgery. Antimicrob Agents Chemother.

[37] Pletz MW, Rau M, Bulitta J, De Roux A, Burkhardt O, Kruse G, Kurowski M, Nord CE, Lode H (2004). Ertapenem pharmacokinetics and impact on intestinal microflora, in comparison to those of ceftriaxone, after multiple dosing in male and female volunteers. Antimicrob Agents Chemother.

[38] Lofmark S, Edlund C, Nord CE (2010). Metronidazole is still the drug of choice for treatment of anaerobic infections. Clin Infect Dis.

[39] Surawicz CM, Brandt LJ, Binion DG, Ananthakrishnan AN, Curry SR, Gilligan PH, McFarland LV, Mellow M, Zuckerbraun BS (2013). Guidelines for diagnosis, treatment, and prevention of *Clostridium difficile* infections. Am J Gastroenterol.

[40] Newton DF, Macfarlane S, Macfarlane GT (2013). Effects of antibiotics on bacterial species composition and metabolic activities in chemostats containing defined populations of human gut microorganisms. Antimicrob Agents Chemother.

[41] Vardakas KZ, Polyzos KA, Patouni K, Rafailidis PI, Samonis G, Falagas ME (2012). Treatment failure and recurrence of *Clostridium difficile* infection following treatment with vancomycin or metronidazole: a systematic review of the evidence. Int J Antimicrob Agents.

[42] Sorg JA, Dineen SS (2009). Laboratory maintenance of *Clostridium difficile*. Curr Protoc Microbiol.

[43] Bartlett JG (2010). *Clostridium difficile*: progress and challenges. Ann NY Acad Sci.

[44] Pepin J (2008). Vancomycin for the treatment of *Clostridium difficile* Infection: for whom is this expensive bullet really magic?. Clin Infect Dis.

[45] Rinttila T, Kassinen A, Malinen E, Krogius L, Palva A (2004). Development of an extensive set of 16S rDNA-targeted primers for quantification of pathogenic and indigenous bacteria in faecal samples by real-time PCR. J Appl Microbiol.

[46] Andersson AF, Lindberg M, Jakobsson H, Backhed F, Nyren P, Engstrand L (2008). Comparative analysis of human gut microbiota by barcoded pyrosequencing. PLoS ONE.

[47] Caporaso JG, Kuczynski J, Stombaugh J, Bittinger K, Bushman FD, Costello EK, Fierer N, Pena AG, Goodrich JK, Gordon JI (2010). QIIME allows analysis of high-throughput community sequencing data. Nat Methods.

[48] Edgar RC (2010). Search and clustering orders of magnitude faster than BLAST. Bioinformatics.

[49] Freeman J, O’Neill FJ, Wilcox MH (2003). Effects of cefotaxime and desacetylcefotaxime upon *Clostridium difficile* proliferation and toxin production in a triple-stage chemostat model of the human gut. J Antimicrob Chemother.

[50] Vohra P, Poxton IR (2011). Comparison of toxin and spore production in clinically relevant strains of *Clostridium difficile*. Microbiology.

