# Supplementary material for: Clostridium difficile colonization and antibiotics response in PolyFermS continuous model mimicking elderly intestinal fermentation
Source: Gut Pathog. 2016 Dec 1;8:63. doi: 10.1186/s13099-016-0144-y (PMC5133761; doi:10.1186/s13099-016-0144-y)
Supplement: Supplementary file 1 — Additional file 1. Effect of ceftriaxone on the microbial composition measured by 454-pyrosequencing on genus level. The microbiota profile in effluents of CR and TR3 of model 2 during the three last days of period B was analyzed by 454-pyrosequencing of the V5-V6 hypervariable regions of the 16S rRNA gene. TR3 was treated with ceftriaxone (=CRO II) during the entire five days of period B. Values < 1% are summarized in the group “others”; uc, unclassified. [file 13099_2016_144_MOESM1_ESM.pptx]

## Slide 1
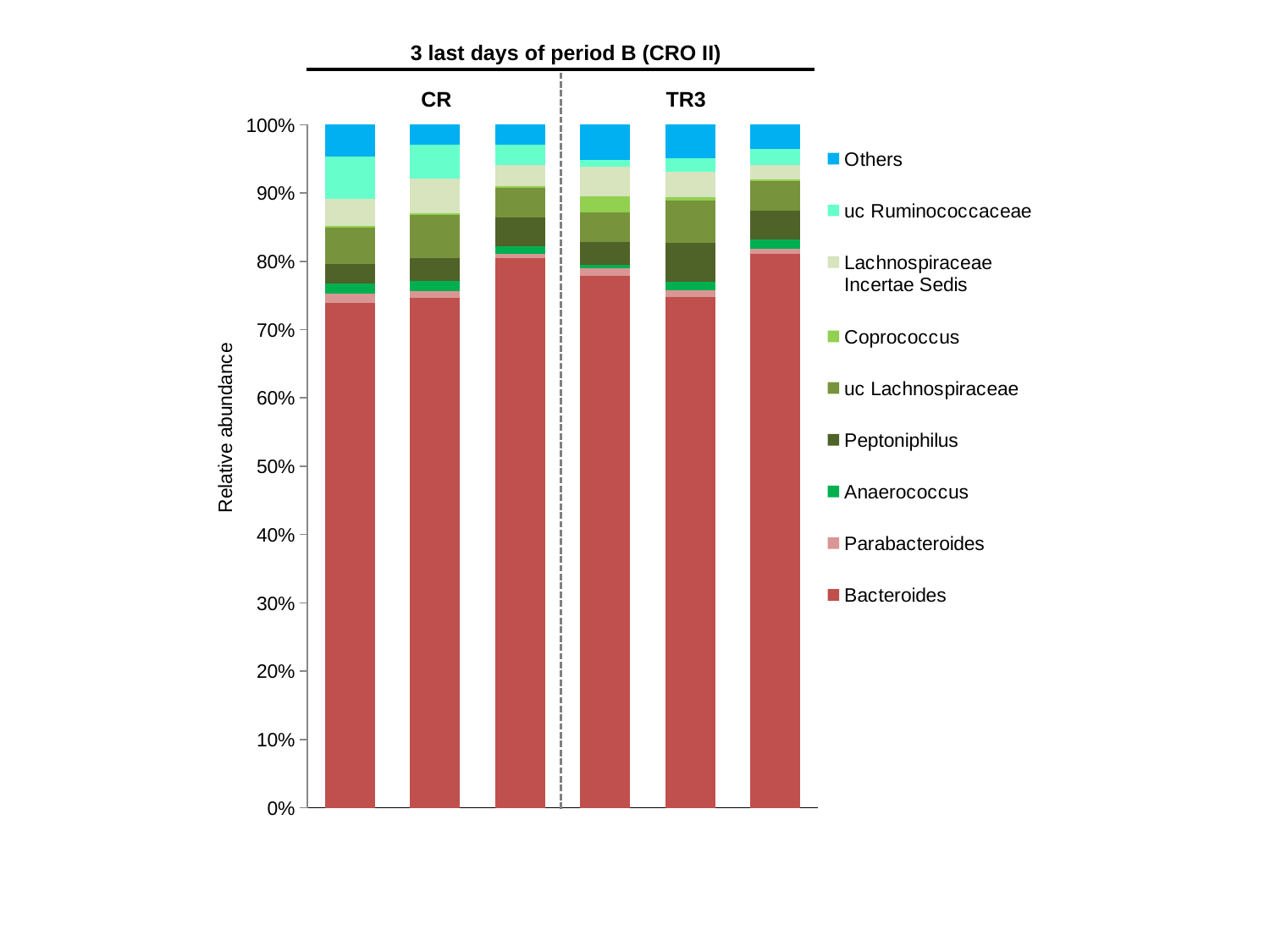

3 last days of period B (CRO II)
CR
TR3
### Chart
| Category | Bacteroides | Parabacteroides | Anaerococcus | Peptoniphilus | uc Lachnospiraceae | Coprococcus | Lachnospiraceae Incertae Sedis | uc Ruminococcaceae | Others |
|---|---|---|---|---|---|---|---|---|---|
| CR_day 3 | 73.88101983 | 1.3597733711 | 1.45042492918 | 2.91218130312 | 5.31444759207 | 0.260623229462 | 3.90934844193 | 6.209631728050001 | 4.6345609065327835 |
| CR_day 4 | 74.353941773 | 0.992258205212 | 1.51564714862 | 3.4020281321600003 | 6.18253189401 | 0.294406280667 | 5.01581070767 | 4.95038708974 | 2.965870679290987 |
| CR_day 5 | 78.6618805464 | 0.528467444411 | 1.1765878951 | 4.09811546515 | 4.177884135999999 | 0.259248180277 | 3.01126732476 | 2.92152757005 | 2.8617010669315874 |
| TR3_day 3 | 77.81425891180001 | 1.18433395872 | 0.445590994371 | 3.34193245779 | 4.32692307692 | 2.39212007505 | 4.36210131332 | 0.996716697936 | 5.089118198896003 |
| TR3_day 4 | 74.7337978178 | 0.9859340081500001 | 1.1831208097800001 | 5.731563034050001 | 6.20481135796 | 0.538977257789 | 3.70711187065 | 1.9850138030800002 | 4.863941106864317 |
| TR3_day 5 | 81.1241425094 | 0.752378844877 | 1.25027660987 | 4.30404956849 | 4.3483071476 | 0.19915910599699999 | 2.1243637973 | 2.36778048241 | 3.507413144500788 |
