# Supplementary figures and images for: Clostridium difficile colonization and antibiotics response in PolyFermS continuous model mimicking elderly intestinal fermentation
Source: Gut Pathog. 2016 Dec 1;8:63. doi: 10.1186/s13099-016-0144-y (PMC5133761; doi:10.1186/s13099-016-0144-y)

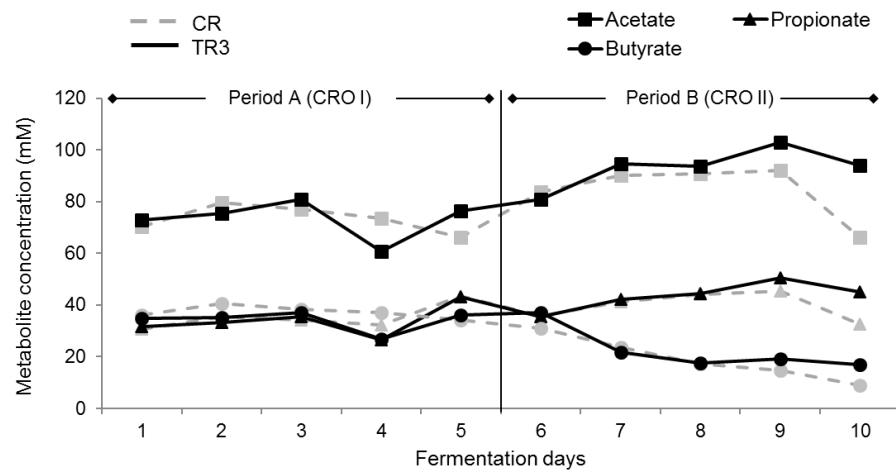

Supplement: Supplementary file 2 — Additional file 2. Daily mean metabolites concentrations in ceftriaxone (=CRO) treated TR3 compared to CR during period A (CRO I) and B (CRO II). [file 13099_2016_144_MOESM2_ESM.pdf]

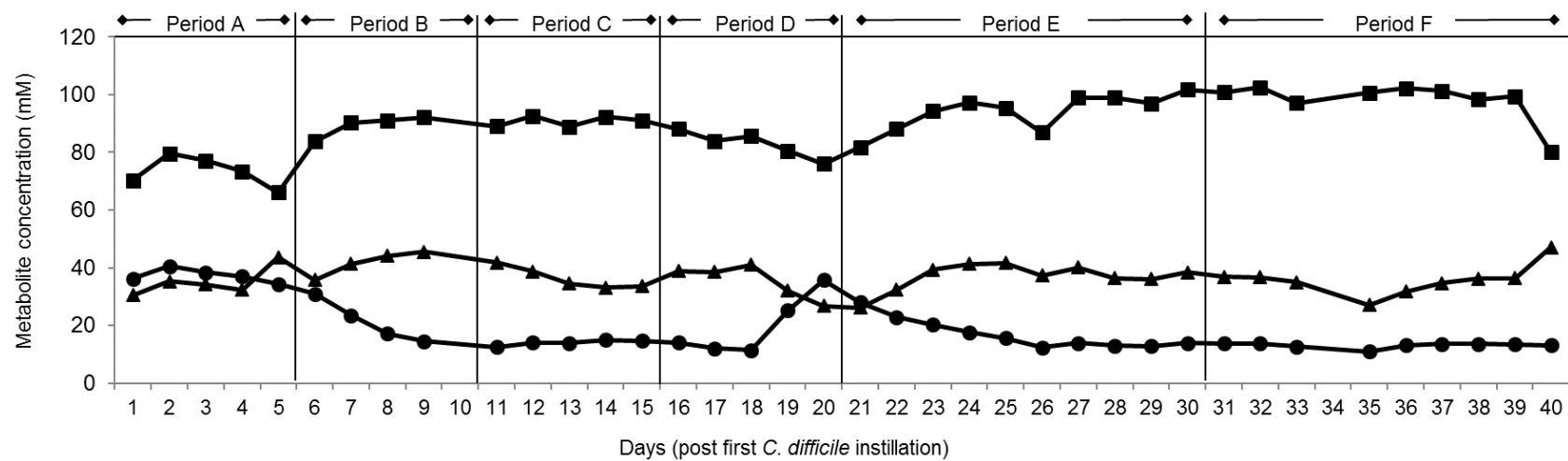

Supplement: Supplementary file 3 — Additional file 3. Daily mean SCFA concentrations in fermentation effluents of CR of model 2 measured by HPLC. From period B on CR was disconnected from continuous feed from IR; (♦) acetate, (■) propionate, (●) butyrate. [file 13099_2016_144_MOESM3_ESM.pdf]

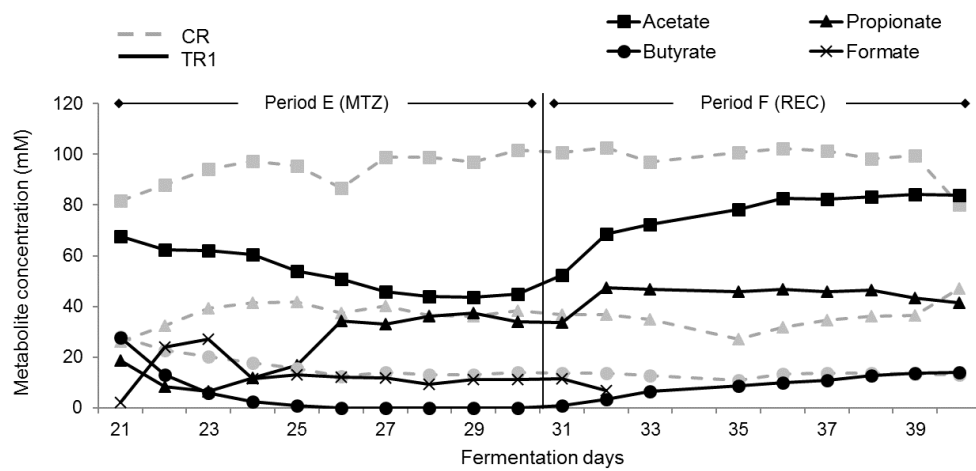

Supplement: Supplementary file 4 — Additional file 4. Daily mean metabolites concentrations in metronidazole (=MTZ) treated TR1 compared to CR during period E and recovery period F (REC). [file 13099_2016_144_MOESM4_ESM.pdf]
