# Supplementary material for: Clostridium difficile colonization and antibiotics response in PolyFermS continuous model mimicking elderly intestinal fermentation
Source: Gut Pathog. 2016 Dec 1;8:63. doi: 10.1186/s13099-016-0144-y (PMC5133761; doi:10.1186/s13099-016-0144-y)
Supplement: Supplementary file 5 — Additional file 5. Effect of metronidazole on the microbial composition measured by 454-pyrosequencing on the phylum and family level. The microbiota profile in reactor effluents of the last three days of period E and F was analyzed by 454-pyrosequencing of the V5-V6 hypervariable regions of the 16S rRNA gene. (A) Relative abundance at phylum level and (B) relative abundance at family level. Values < 1% are summarized in the group “others”. MTZ, metronidazole; REC, recovery; uc, unclassified. [file 13099_2016_144_MOESM5_ESM.pptx]

## Slide 1
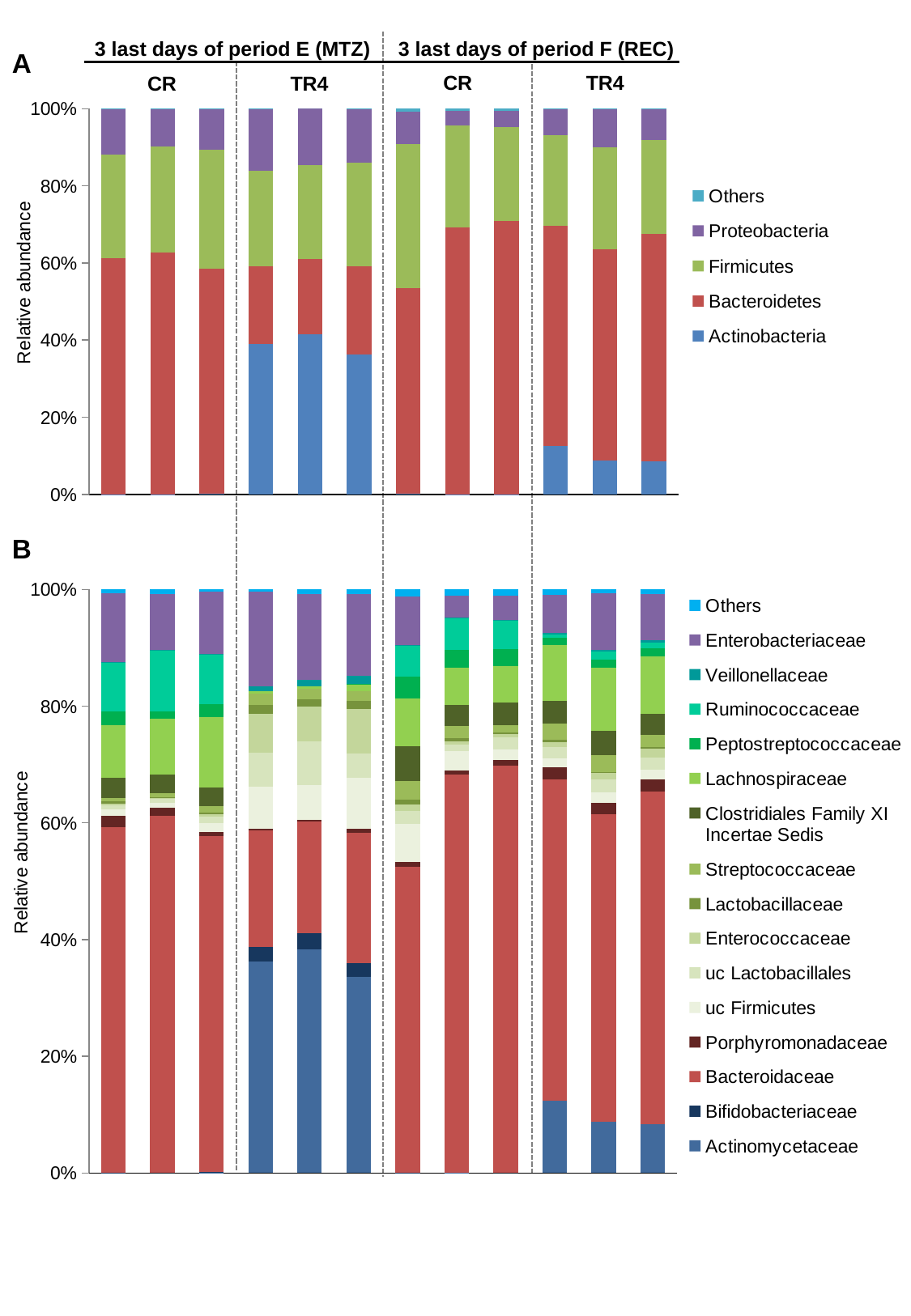

3 last days of period E (MTZ)
3 last days of period F (REC)
A
CR
TR4
CR
TR4
B
### Chart
| Category | Actinobacteria | Bacteroidetes | Firmicutes | Proteobacteria | Others |
|---|---|---|---|---|---|
### Chart
| Category | Actinomycetaceae | Bifidobacteriaceae | Bacteroidaceae | Porphyromonadaceae | uc Firmicutes | uc Lactobacillales | Enterococcaceae | Lactobacillaceae | Streptococcaceae | Clostridiales Family XI Incertae Sedis | Lachnospiraceae | Peptostreptococcaceae | Ruminococcaceae | Veillonellaceae | Enterobacteriaceae | Others |
|---|---|---|---|---|---|---|---|---|---|---|---|---|---|---|---|---|
